# Machine-learning-aided analysis of relationship between crystal defects and macroscopic mechanical properties of TWIP steel

**DOI:** 10.1038/s41598-025-99319-8

**Published:** 2025-04-25

**Authors:** Marino Tanaka, Kai Sasaki, Jesada Punyafu, Mayu Muramatsu, Mitsuhiro Murayama

**Affiliations:** 1 Department of Science for Open and Environmental Systems, Graduate School of Keio University, 3-14-1, Yokohama, Kanagawa 233-8522 Japan; 2 Institute for Materials Chemistry and Engineering, Kyushu University, Fukuoka, 816-8580 Japan; 3 Department of Mechanical Engineering, Keio University, 3-14-1, Yokohama, Kanagawa 233-8522 Japan; 4 Department of Materials Science and Engineering, Virginia Tech, Blacksburg, VA 24061 USA

**Keywords:** TEM, Dislocation, Stacking fault, Machine learning, TWIP steel, Mechanical properties, Scientific data

## Abstract

Establishing efficient methods to obtain quantitative data on crystal defect evolution is vital for understanding material properties. Dynamic Transmission Electron Microscopy (TEM) captures crystal defects in materials undergoing plastic deformation, generating vast datasets with high temporal and spatial resolution. However, manual analysis of these images is labor-intensive, and automated, unbiased analysis remains a challenge. In this study, we developed a U-net-based machine learning approach to analyze TEM videos of crystal defect evolution in Twinning-Induced Plasticity (TWIP) steels with different grain sizes. The method overcame challenges like field-of-view translation and nonuniform defect motion. This approach quantitatively measured defect evolution as a function of time and strain with the same temporal resolution as the original videos, detecting even minor changes with high accuracy. We use this technique to quantitatively reveal the switch of the dominant plastic deformation mechanism with grain size and the relaxation of elastic strain due to the rapid increase in stacking faults. Our results validate the use of U-net models for efficient semantic segmentation of TEM videos, enabling accurate quantitative analysis. This work advances TEM video analysis and provides new insights into the deformation mechanisms of materials.

## Introduction

Twinning-induced plasticity (TWIP) steels have been widely researched because of their high strength and large ductility balance^1–3^. TWIP steels generally have a low stacking fault energy and the twinning deformation occurs at relatively small strains. This phenomenon is known as TWIP effect^4,5^ and is responsible for the superior mechanical properties of TWIP steels.

Fe-22Mn-0.6 C is one of the typical high-strength type TWIP steels and has been attracting both scientific and engineering attention^6–8^, for example, serration^8–10^ on its stress-strain curve is a good case in point. A number of research have been carried out to reveal the mechanism of occurrence of serration. Initially, serration was explained by the dynamic strain aging, i.e., the temporal arrest of mobile dislocations by C atoms^11,12^. However, considering the diffusion rate of carbon at room temperature and the serration cycle or observed dislocation velocity, dynamic strain aging is unlikely to explain the non-uniform deformation, then various other mechanisms have been proposed and discussed so far^13,14^. In addition to the mechanism, factors affecting the macroscopic mechanical response such as the solute-atomic concentration^15,16^, deformation temperature^17,18^, strain rate^19–23^, and grain size^24,25^, have also been investigated.

The grain size dependence of the deformation mechanism of TWIP steels is also a major research subject relevant to the strength – ductility balance improvement. Transmission electron microscopy (TEM) is widely used as a technique that enables the direct observation of dislocations and stacking faults^4,26–29^. Using TEM, Hung et al.^30^ revealed that the deformation mechanism in an Fe-31Mn-3Al-3Si TWIP steel makes a transition from slip to twinning as the grain size decreases. When the grain size is larger than 1 μm, the dislocations are more significant than the stacking faults. When the grain size becomes smaller than 1 μm, the stacking faults nucleated and grown from the grain boundaries are more significant than the dislocations. A similar grain size dependence was also found in an Fe-22Mn-0.6 C TWIP steel by Punyafu et al.^9^.

From the viewpoint of quantitative observation, however, both Hung et al.^30^ and Punyafu et al.^9^ leave much to be improved. First, Hung’s study^30^ performed only a qualitative evaluation and they didn’t evaluate the amounts of dislocations or stacking faults. Punyafu’s study^9^ made some progress in terms of quantitative assessment over Hung’s study^30^ but their measurements were manually implemented. They detected dislocations and stacking faults visually and counted the number of grains that contained dislocations and stacking faults. A common problem with these studies was that they observed crystal defects at only limited strain values such as three points near the macroscopic yield point. As such, observations by static TEM were often limited to a qualitative evaluation. Even when quantitative measurements were conducted, those were mostly manually done. In consequence, the vast majority of conventional quantitative measurements of defects or microstructural features by static TEM are restricted by the labor-intensive manual counting operations, and can only evaluate crystal defects at relatively small, limited number of cases.

To measure crystal defect evolution under continuous loading, dynamic TEM observation is essential. Recently, techniques and methodologies to capture moving images of crystal defects in materials under deformation have been progressed^31–36^. However, it remains challenging to apply a conventional manual evaluation method to every frame of video because of its high cost of the work. Additionally, the discrimination by the human eyes generates the labeled data with no certain definition.

To solve this problem, we conducted a machine-learning-aided analysis. Since properly trained machine learning can extract unbiased features from data sets, it has been applied to a wide range of research fields, and the research on materials is not an exception. For example, some studies report the prediction of new multiphase high-entropy alloys^37^, the prediction of crystallographic orientation evolution of polycrystalline materials^38^, the identification of defects in amorphous alloys^39^, and the prediction of defects and their types in friction stir welding^40^. A similar example to our work in which machine learning is applied to dislocations is the work by Sasaki et al.^41^. They have developed a framework to measure the velocities of individual dislocations from TEM video using U-net^42^ - based machine learning. The U-net is one of the major machine-learning models used for semantic segmentation which is the task of labeling pixels in an image^42–44^. U-net consists of convolutional layers and deconvolutional layers and skip connections connecting them to realize highly accurate semantic segmentation. In Sasaki’s study^41^, they successfully classified the each of image pixels into two classes, i.e., dislocations and backgrounds.

In this study, we measured the amounts of dislocations and stacking faults in the continuous strain effectively and quantitatively using U-net. It is considered that a three-class classification by the use of U-net into the dislocations, stacking faults, and background would allow us to investigate the grain size dependence of the deformation mechanism and the relationship between the yield point and those crystal defects.

## Method of experiment and machine learning

**Fig. 1:**
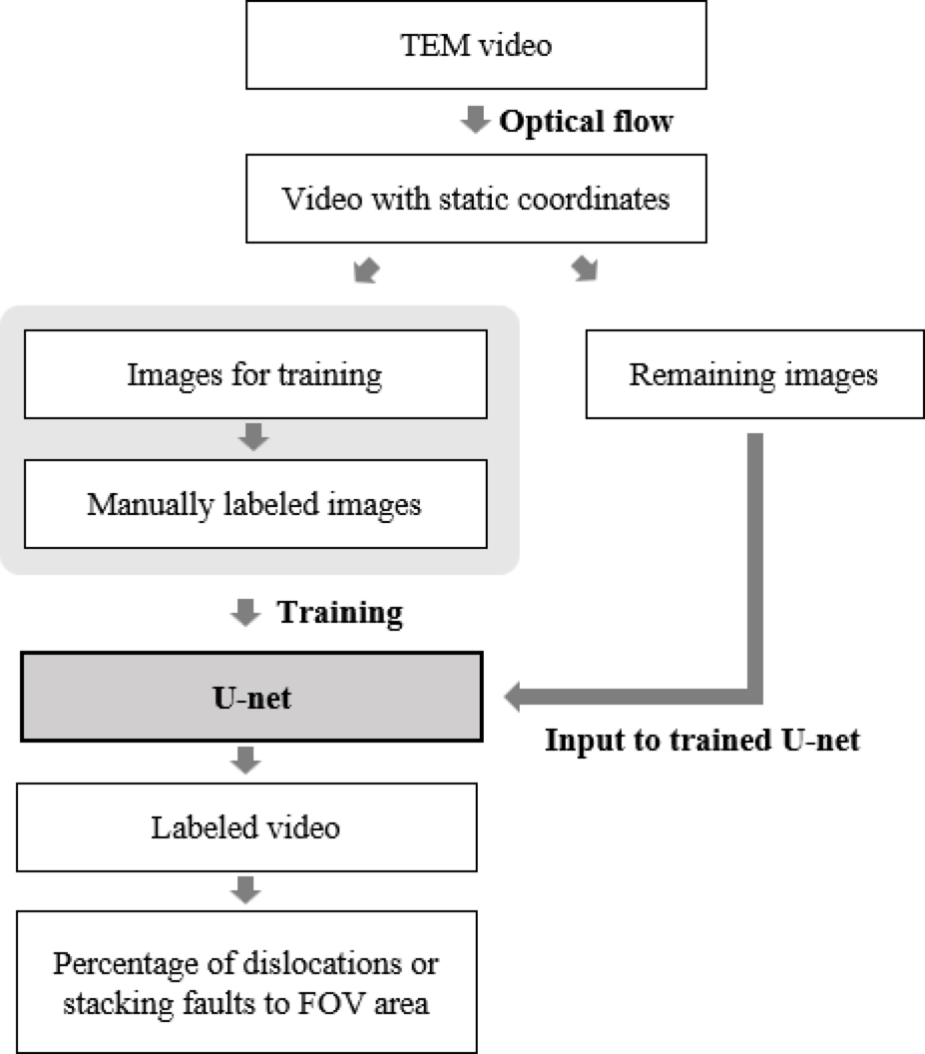
Conceptual diagram of the method to measure the percentage of dislocations or stacking faults to FOV area.

Amounts of dislocations and stacking faults in a specific field-of-view (FOV) area of a TEM video were calculated from the number of pixels following the flow shown in Fig. 1, which is based on the framework developed by Sasaki et al. (cf., Fig. 5 in^41^). Three TEM videos exhibiting the plastic deformation behavior of an Fe-22Mn-0.6 C TWIP steel with three different average grain sizes of 0.86 μm, 2 μm, and 8.4 μm, were examined. The manufacturing processes of the three specimens involved multistep cold-rolling and annealing processes, followed by post-heat treatment to control the desired grain size. Detailed process is reported in^45^. In the experiments, a thin foil of TWIP steel was subjected to a forced displacement at a constant rate in a TEM with a specially designed specimen holder. Specimens for this in-situ TEM deformation observation were sliced from as-annealed sheets by a diamond wire saw and then mechanically polished to 100 μm thick. Thinning to electron transparency was achieved by using a cryo-ion slicer (JEOL IB-09060CIS) at -150 °C with an applied voltage of 6 kV for Ar ion beam. The foil having an electron transparent area was then fixed on a cartridge-type blade for Strain and Tomography (SATO) Holder^31^ and continuously deformed at room temperature inside a JEOL ARM-200 F TEM operated at 200 kV. In-situ TEM deformation experiments were performed under the conditions in Table 1. All videos were recorded at 20 frames per second. Multiple videos were recorded for each of grain size, then the best quality one was used for this analysis.

**Table 1:** Conditions of plastic deformation experiments. NOTE: “initial strain” means the strain value of the specimen at the beginning of the video.

Grain size	0.86 μm	2 μm	8.4 μm
Material	Fe-22Mn-0.6 C		
Initial strain	$\:9.99\times\:{10}^{-3}$	$\:3.40\times\:{10}^{-2}$	$\:1.14\times\:{10}^{-2}$
Strain rate (s^-1^)	$\:5.00\times\:{10}^{-5}$	$\:2.50\times\:{10}^{-5}$	$\:1.00\times\:{10}^{-4}$

**Fig. 2:**
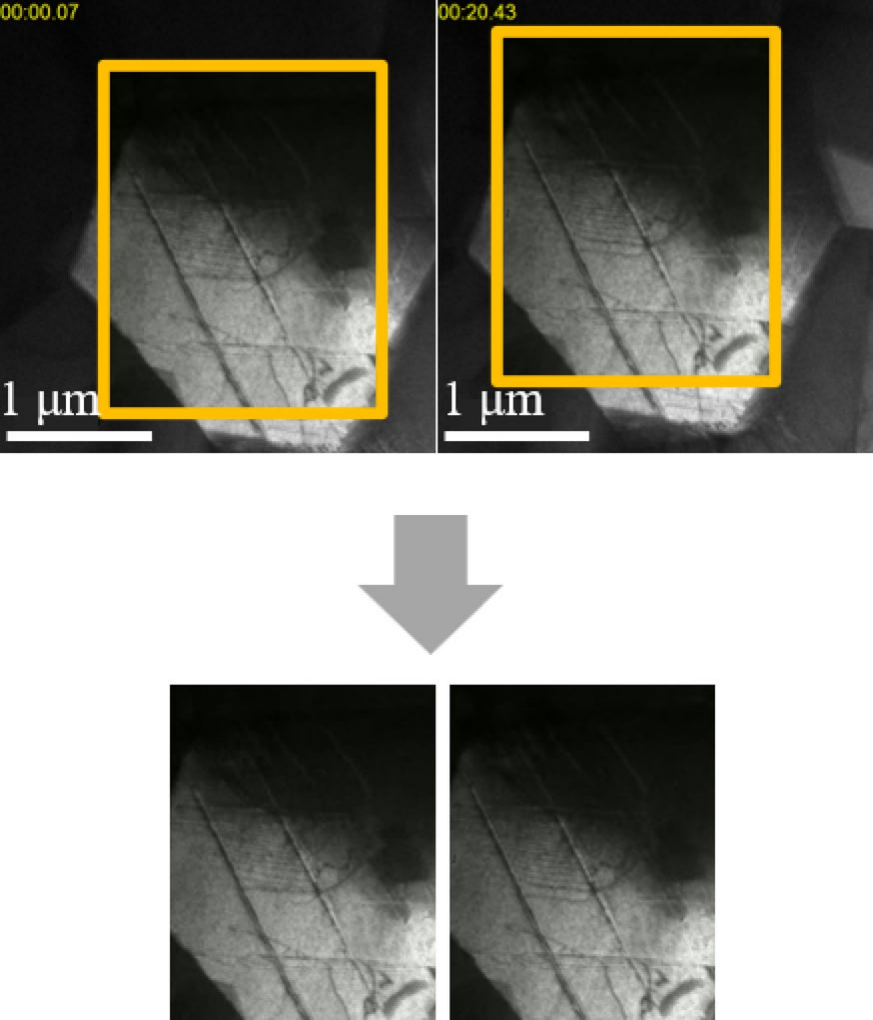
Fixation of the FOV in TEM videos by an optical flow estimation. The original frames with moving FOV were converted into the images in which the same area of the specimen was captured. This process was applied to all of the frames.

During in-situ deformation experiments, dislocations glide and deformation twins grow under applied external stresses; these sometimes extend beyond the camera frame, i.e., the area being recorded. External forces applied to cause plastic deformation also sporadically move the sample itself. Therefore, the camera frame, or Field of View (FOV), has to be changed on each occasion to retain a particular set of moving defects in sight and/or to capture wide-ranging phenomena for statistical relevancy. One could think of it like a video camera panning to keep a walking subject in frame as their background changes. If not taking this point into account, the observed increases or decreases in dislocations and stacking faults in a video could be responsible to the FOV shift rather than the microstructure response. Therefore, to avoid such possible miscount due to the camera position variation, the FOV in each of video frames were trimmed to make the background of two adjoining frames uniform by an optical flow estimation^46–48^ so that the FOV would be consistent throughout an analysis. The method of fixing the FOV is shown in Fig. 2. Using the optical flow, some specific feature points were tracked. First, the optical flow was calculated for all frames, and a trimming area was determined based on the frames with the largest and smallest displacements. Then, for each frame, by calculating the displacement of the feature point relative to the immediately preceding frame and shifting the trimmed area according to the calculated displacement, the original videos were converted to the ones in which the same area of the specimen was captured for all frames.

**Fig. 3:**
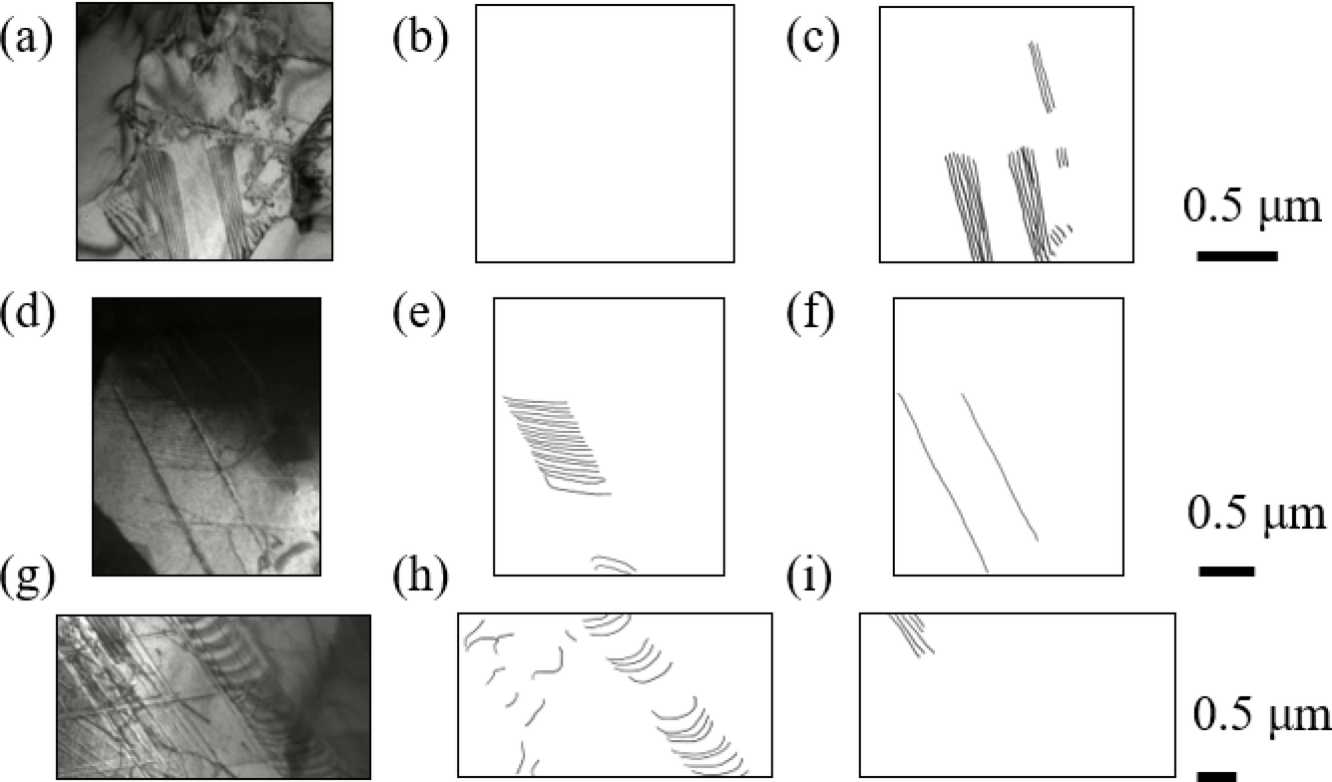
(**a**,**d**,**e**) several characteristic frames selected from in-situ deformation TEM videos and their mask images identifying (**b**,**e**,**h**) dislocations and (**c**,**f**,**i**) stacking faults. Note: (**a**–**c**) the 0.86 μm grains. (**d**,**e**) the 2 μm grains. (**g**–**i**) the 8.4 μm grains.

After the conversion from each original video to a video with static coordinates, mask images were created for the frames of the video. As shown in Fig. 3, we prepared two mask images. In the first mask image, each pixel was labeled either as dislocation or background, while in the second mask image, each pixel was labeled either as stacking fault or background. Then those were used as teacher data of machine learning. The frames to be used as teacher data were basically selected evenly throughout the time series, whereas some additional frames were chosen from a period when changes were drastic thus the training of machine learning was challenging. For example, for the 8.4 μm grains, one training data frame was selected from every 20 frames at first. In other words, the training data was at 1 fps because the frame rate of the original video was 20 fps. The dislocation moved quickly after the 180th frame for this grain size and 1fps would be insufficient to provide enough similar teacher data. Therefore, training data were selected every 2 to 5 frames from the 180th frame onward.

**Fig. 4:**
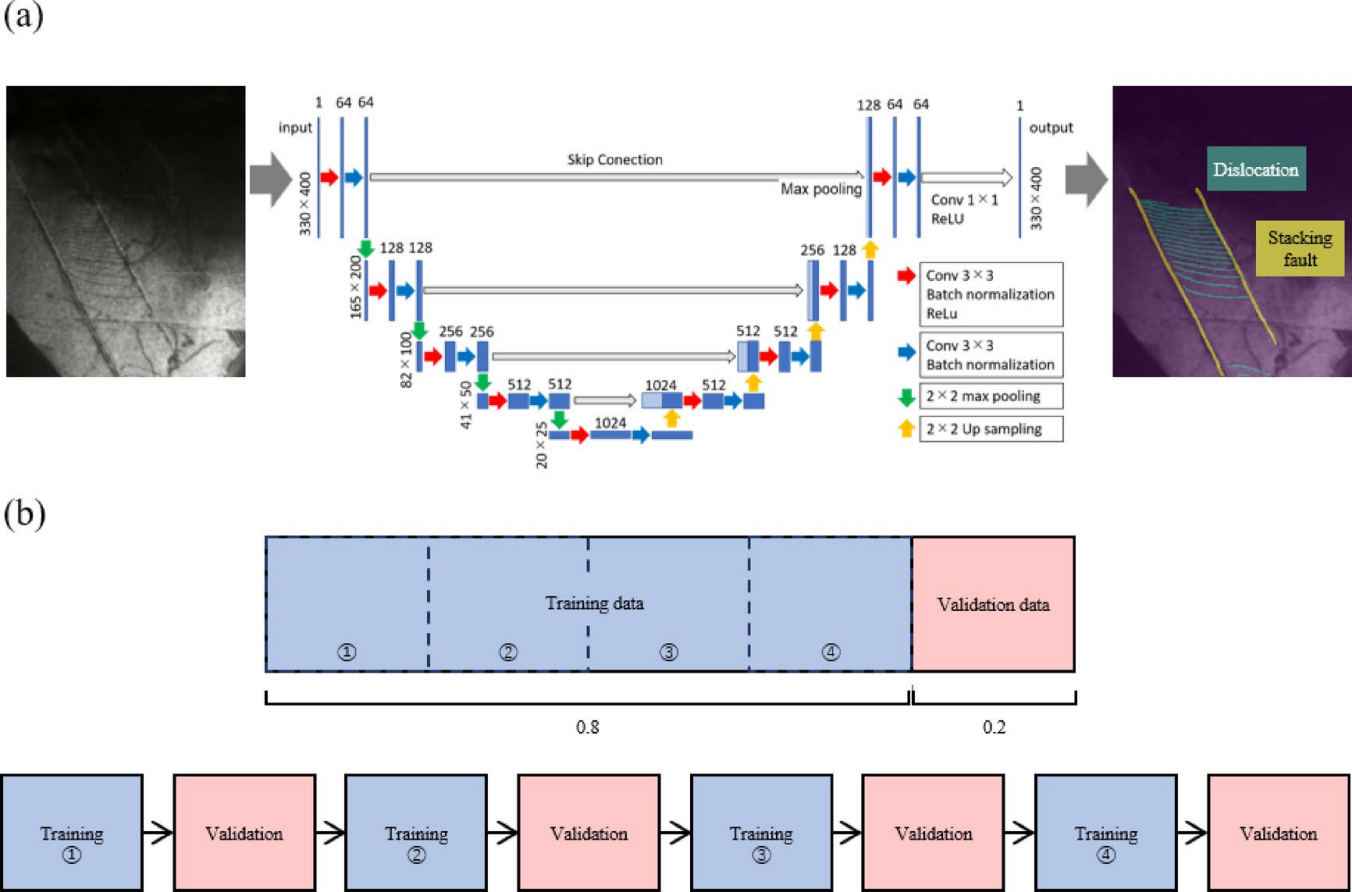
(**a**) The architecture of the U-net (for 330 × 400 pixels) and input/output images. (**b**) Schematics show the sequence of choosing training data and validation data as well as the training flow. 20% of teacher data were used for validation and 80% of teacher data were used for training. In 1 epoch of training, every time a quarter of training was completed, validation is performed using all of the validation data.

To make mask images, the dislocation and stacking fault were respectively labeled. Sessile dislocations were not labeled because they didn’t contribute to the increase of dislocations. Note that only stacking faults were labeled for the 0.86 μm grains since no dislocation was observed. Based on the original images and corresponding mask images, three U-nets were trained for three videos of different grain-sized specimens. Figure 4a shows the architecture of U-net, and input and output images in this study. The training flow is shown in Fig. 4b. During the training of each epoch, a hold-out validation was performed to verify the accuracy of the machine learning model. The dataset was divided into training and validation sets, where 20% of the dataset was reserved for validation. This allowed for an unbiased evaluation of the model’s performance on unseen data during training. In the training, the prediction outputs by the U-net were compared with the mask images to calculate the loss, and the U-net parameters were updated based on the value of loss. In the validation process, the loss was calculated in the same way as the one in training, and the learning rate was adjusted based on the value of the loss. The ReduceLROnPlateau was used as the scheduler to control the learning rate adjustment, and the rate became 0.1 times smaller than the original when the loss didn’t decrease for two epochs. Finally, by inputting the remaining frames that were not used for training, the video was automatically labeled as the output from the trained U-net. The output was an array with the values of 0 for background, 1 for dislocations, and 2 for stacking faults. The number of pixels labeled with dislocations and stacking faults was calculated from the output array and divided by the total number of pixels in the image. In this way, the percentages of dislocations and stacking faults to the FOV area were measured for each video in continuous strain.

This method offers several advantages: (1) It takes less cost of work than the conventional method. Even though the labeling process still requires some labor, it is significantly reduced compared to conventional methods: for the video of 2 μm grains, only 63 mask images enabled labeling 2360 frames automatically and correctly. (2) It enables the labeling of a large series of TEM images based on a unified standard. In other words, even though labeling criteria by humans have some degree of variation from frame to frame, machine learning learns them comprehensively and labels all frames based on certain uniform criteria. Also, any potential errors in labeling would be systematic and correctable. (3) The continuous changes (time trend) in dislocations and stacking faults can be measured because a large number of frames can be handled easily using machine learning thus no need to omit frames from the analysis.

As for the potential for generalization of the proposed method to other metallic materials, since machine learning itself does not take into account material mechanics derived from crystal structures, etc., but learns image features, the results are considered to be independent of the material. Therefore, as long as the shape of the dislocations or stacking faults are not significantly different between frames of a single TEM video, we believe that this approach can be applied to other materials.

## Results

**Fig. 5:**
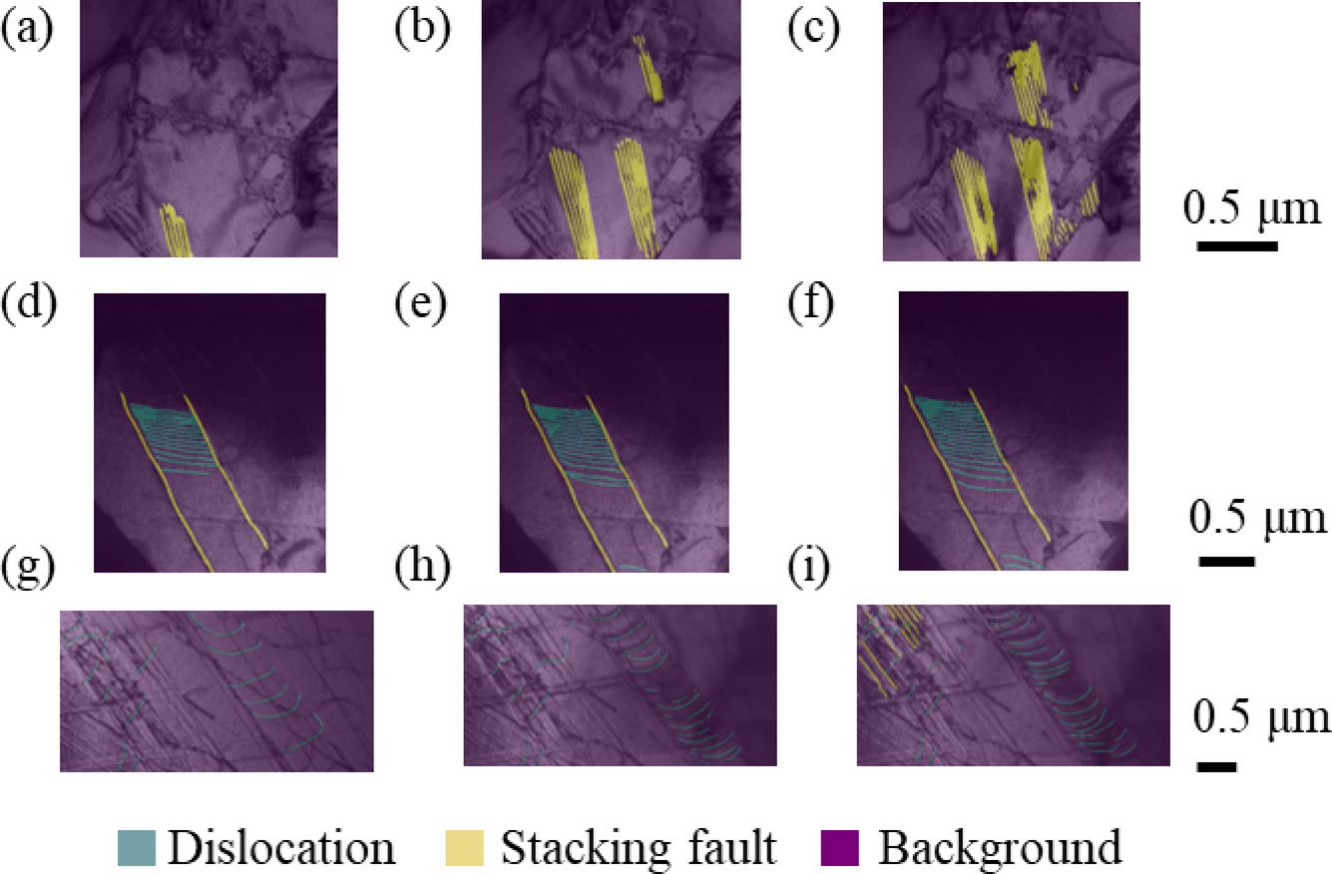
Images outputted from U-net demonstrating how dislocations and stacking faults change as the strain $\:\epsilon\:$ changes. (**a**–**c**) the 0.86 μm grains. (**d**,**e**) the 2 μm grains. (**g**,**i**) the 8.4 μm grains. Each image was taken under the following strains; (**a**) $\:\epsilon\:=1.024\times\:{10}^{-2}$ (**b**) $\:\epsilon\:=1.050\times\:{10}^{-2}$ (**c**) $\:\epsilon\:=1.110\times\:{10}^{-2}$ (**d**) $\:\epsilon\:=3.446\times\:{10}^{-2}$ (**e**) $\:\epsilon\:=3.501\times\:{10}^{-2}$ (**f**) $\:\epsilon\:=3.670\times\:{10}^{-2}$ (**g**) $\:\epsilon\:=1.162\times\:{10}^{-2}$ (**h**) $\:\epsilon\:=1.237\times\:{10}^{-2}$ (**i**) $\:\epsilon\:=1.256\times\:{10}^{-2}$.

The performance of the model on the validation set was measured using the F-score, which is one of the indices used in machine learning to evaluate a two-class classification problem^49^. Benchmark results showed that F-scores improved with each epoch, with convergence values of F-scores exceeding 0.7 for each particle size. Figure 5 shows some of the images automatically labeled by the trained U-net. Each pixel seems to be correctly labeled.

**Fig. 6:**
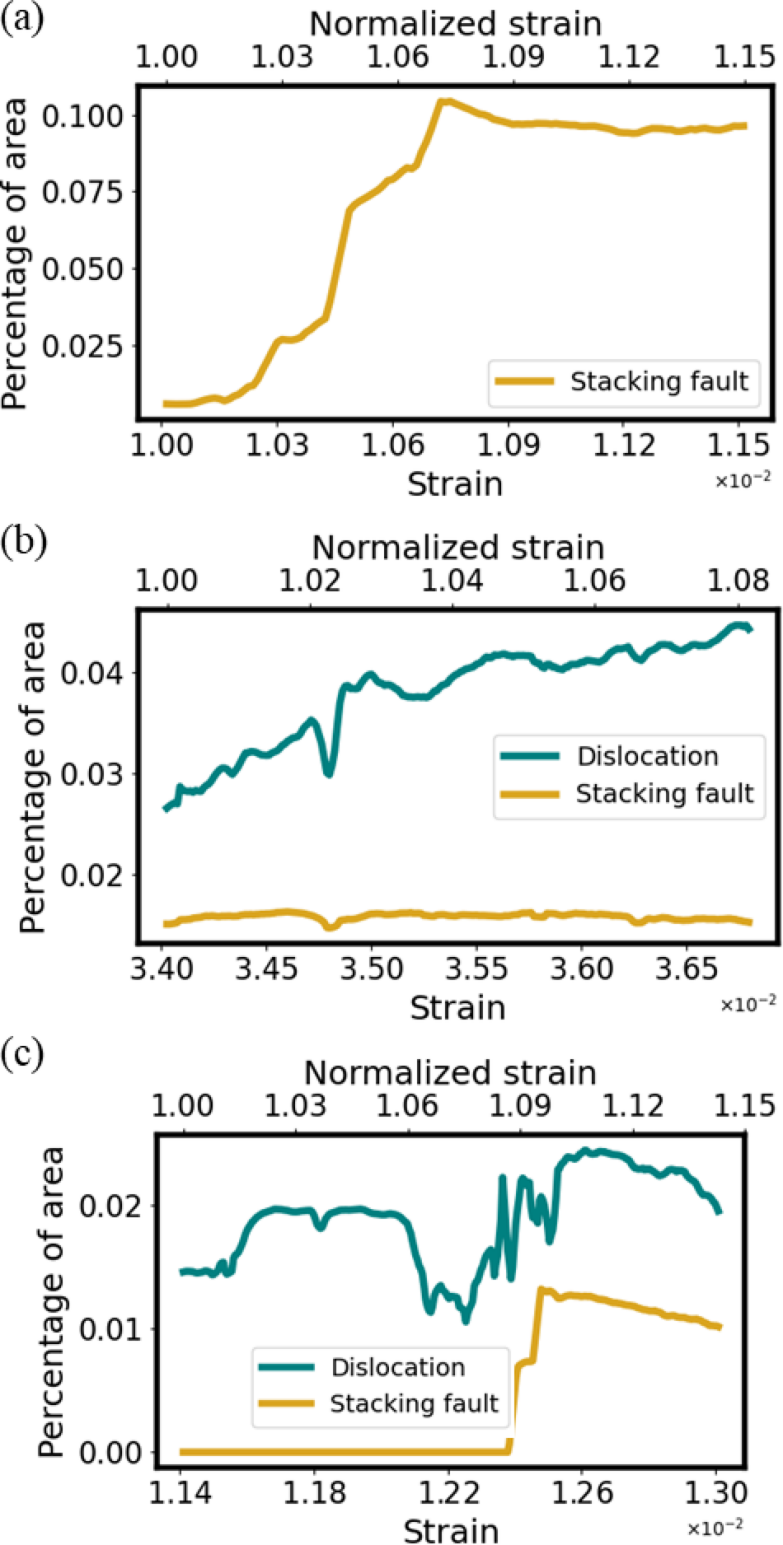
Changes in the percentage of dislocations and stacking faults in the FOV as a function of strain; (**a**) the 0.86 μm grains, (**b**) the 2 μm grains, (**c**) the 8.4 μm grains.

Figure 6a–c show the changes in the area ratio (percentage) of dislocations and stacking faults in the FOV as a function of strain. The upper x-axis of the figure shows the strain values normalized by the initial strain of each specimen.

In the result of the 0.86 μm grains (Fig. 6a), stacking fault area actively increased while no dislocation area was observed. It has been reported in various metals and alloys that dislocations are difficult to form from grain interior Frank-Read sources when the grain size becomes smaller than 1 μm^50–52^. Therefore, the fact that no dislocation was detected in the 0.86 μm grains even before stacking faults formation by machine learning appears to be reasonable. While the strain is between $\:1.07\times\:{10}^{-2}$ and $\:1.08\times\:{10}^{-2}$, the amount of stacking fault decreases. In the TEM video’s corresponding time period, some of the stacking faults actually being disappeared. Possible explanations of this phenomenon would be, trailing partial dislocations were emitted from grain boundaries then caught up with their leading partial dislocations when the local stress at these boundaries reached the starting stress of the trailing partials, or these stacking faults transformed to deformation twins as previously observed by an in-situ TEM study and lost their image contrast^53^. Using machine learning, even such a trivial change was able to be measured.

In the result for the 2 μm grains (Fig. 6b), the dislocations increased though the stacking faults didn’t increase significantly. In the result for the 8.4 μm grains (Fig. 6c), an increase in stacking faults was observed when the strain became larger than 1.23 × 10^− 2^.

On the other hand, dislocations increased and decreased actively when the strain exceeded 1.21 × 10^−2^ and the final percentage of dislocation increased compared to the beginning of the TEM video. When the number of dislocations changed drastically in Fig. 6c, the corresponding timeframe of the TEM video showed that large number of dislocations moved rapidly and simultaneously. Thus, it was considered to be dislocation avalanches^54^ or the release of the dislocation group from pinning by carbon atoms as the local stress achieved its criteria^55^. The cause of the increase and decrease is considered to be that the machine learning model not completely detected dislocations. While this period, and the number of dislocations in the field of view continues to increase. By improving the accuracy of the machine learning model, such as by increasing the training data, this increase or decrease can be eliminated and the rapid increase in dislocations can be captured more accurately. Although it was not able to completely detect this rapid increase in dislocations this time, it is worthwhile to be able to automatically label thousands of images at a small cost and quantitatively evaluate the amount of dislocations and stacking faults with a certain degree of accuracy.

## Discussion

### Grain size dependence of deformation mechanism

**Fig. 7:**
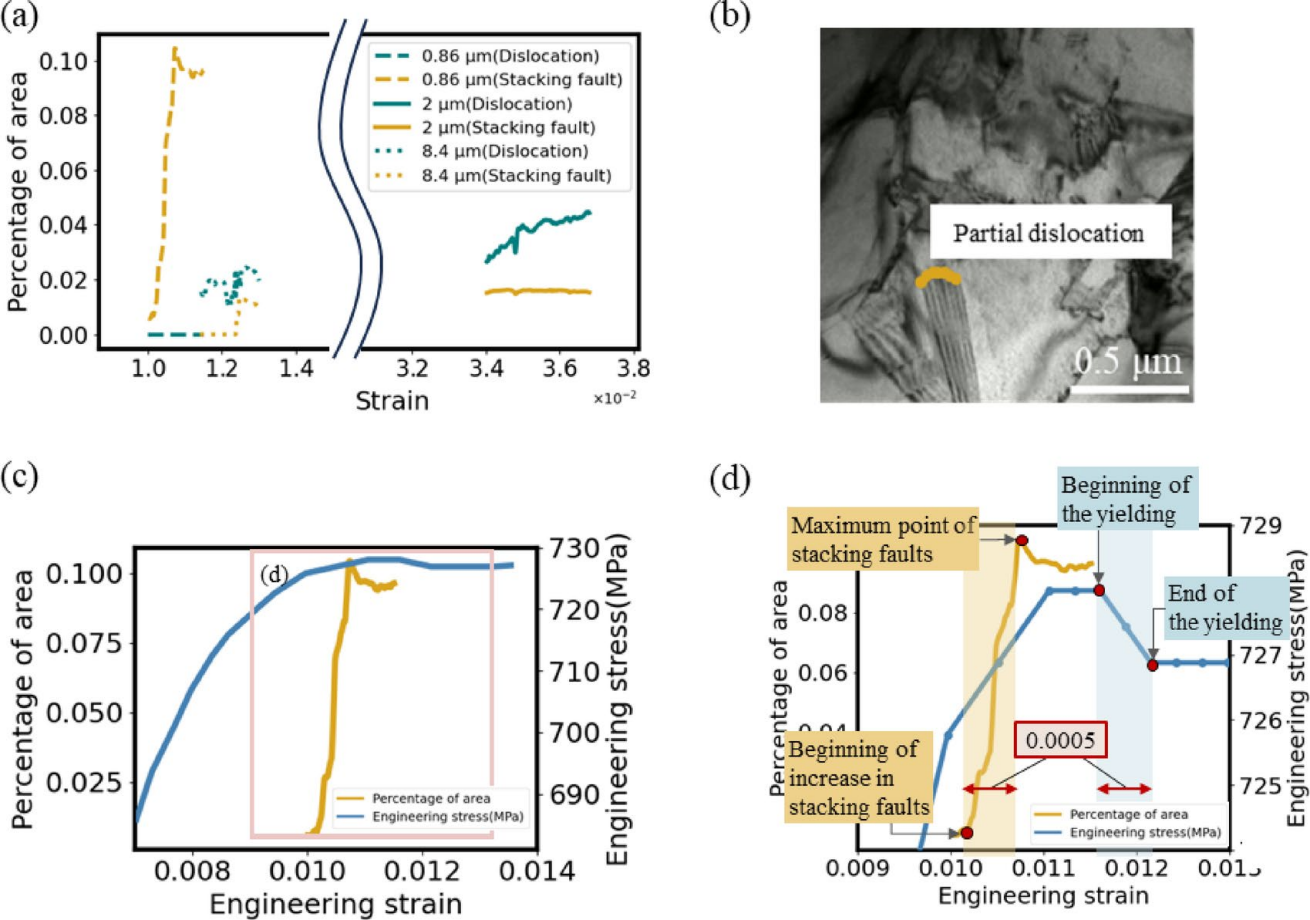
(**a**) Changes in the percentage of dislocations and stacking faults in the FOV to strain of all grain sizes. (**b**) Leading partial dislocations that from stacking faults. (**c**) Percentage of stacking faults in the FOV of 0.86 grains and stress-strain curve. The strain value in which stacking faults increase almost coincides with that of the yield point. (**d**) The comparison of the increasing rate of stacking faults with the slope of the stress-strain curve near the yield point. The strain increment from the upper yield point to the lower yield point and that from the start to the maximum points of the stacking faults increment coincide with each other.

Figure 7a shows the percentages of the dislocations and stacking faults in the FOV as a function of applied strain for all grain sizes. The value of dislocations for the 0.86 μm grains is zero because no dislocation was observed and only stacking faults were observed. On the other hand, in the 2 μm and 8.4 μm grains, dislocations were more pronounced than stacking faults. These observations support the widespread hypothesis, i.e., initiating plastic deformation by dislocation slip in ultrafine grains (typically less than 1 μm) is unlikely the case because the pre-existing dislocations in such small grains become negligible^56^. It means that as grain size decreases, the deformation mechanism switches from the slip deformation due to dislocations to the twinning deformation beginning with stacking fault formation as a precursor phenomenon. The current observation is also consistent with previous grain size dependence studies in ultrafine-grained (UFG) materials including TWIP steels (e.g^9,30,50–52^). This consistency in structural defect identification and categorization validated that our ML-based pipeline has been well dependable as an automated quantitative measurement approach.

### Difference of velocity of partial and perfect dislocations

Regarding Fig. 7a, we compare the velocity of partial and perfect dislocations. By definition, a stacking fault exists between a leading partial dislocation and a trailing partial dislocation. In other words, the stacking faults disappeared from the region where the trailing partial dislocation has passed through. In the results of the 0.86 μm grains, trailing partial dislocations are considered to have not been emitted from the grain boundary since each stacking fault is not annihilated while the strain is less than $\:1.07\times\:{10}^{-2}$. Therefore, the growth rate of the stacking fault in the period can be regarded as the velocity of leading partial dislocations (Fig. 7b). Comparing the percentages of dislocations and stacking faults in the FOV for each grain size in Fig. 7a, the increase in stacking faults for the 0.86 μm grains is significant. Therefore, the partial dislocations that form stacking faults in the 0.86 μm grains are considered to move faster than the perfect dislocations in other two grain sizes. A possible explanation for the faster velocity of the leading partial dislocations in the 0.86 μm grains is that the interaction between carbon atoms and partial dislocations is smaller than that between carbon atoms and perfect dislocations by assuming carbon atoms – dislocation interactions occur in this alloy system which implied by the serration on macroscopic stress-strain curve.

### Increase of stacking fault and discontinuous yielding in 0.86 μm grains

The grain size 0.86 μm specimen exhibits discontinuous yielding behavior which was not the case in the grain size 2 μm specimen^9^. The evolution of the stacking fault in the 0.86 μm specimen (obtained by this study) along with its stress-strain curve (taken from^9^), is shown in Fig. 7c. The strain value, at which the number of stacking faults showed a sudden increase, almost coincides with the yield point. This is consistent with the results of UFG Fe-31Mn-3Al-3Si TWIP steel by Bai et al.^50^. A more precise evaluation around the yield point in Fig. 7d shows that the yielding occurred shortly after the increase in stacking faults has started. This can be attributed to differences in the Schmidt factor and activation stress of individual grains. The activation stress is the stress required for the stacking faults to generate. Since a large number of grains exist outside of the area of observation and each has its own Schmidt factor^57^ and activation stress, the observed rapid increase phenomenon would occur with differently-timed in other grains, thus the macroscopic yielding representing the collective behavior of individual grains occurs only after a certain amount of time.

Figure 7d also shows that the strain increment from the upper yield point to the lower yield point and that from the starting point of the sudden increase until reaching the maximum of stacking faults were both nearly the same value, i.e., 5 × 10^− 4^. Therefore, the rapid increase in stacking fault is related to discontinuous yielding. It is considered that a rapid increase in stacking faults releases the elastic energy stored in the specimen^58,59^ and leads to macroscopic yielding. A similar correlation was indicated in other UFG TWIP steel^30^.

## Conclusions

In summary, TEM videos captured the plastic deformation behavior of Fe-22Mn-0.6 C TWIP steel were semantically segmented by U-net to efficiently and quantitatively measure the continuous changes in the number of dislocations and stacking faults, the plastic strain carriers. The method enables us to handle a large number of individual frames in TEM videos, which has been difficult due to the cost and poor reproducibility of naked-eye-based analyses. By our proposed method, a statistically relevant volume data to discuss the trend in the evolution of dislocations and stacking faults was obtained from a single video automatically. The measurement results confirm the switch in the deformation mechanism depending on the grain size. Furthermore, the increase in stacking faults in the 0.86 μm grains is more remarkable compared to the other two grain sizes, suggesting partial dislocations emitted from grain boundaries move faster than the perfect dislocations observed in other grain sizes. The rapid increase in stacking faults in the 0.86 μm grains is closely related to the discontinuous yielding behavior in ultrafine-grained metallic materials. Although these phenomena have already been discussed qualitatively, we have now shown that macroscopic mechanical properties can be quantitatively described in terms of changes in the number density of crystal defects or plastic strain carriers.

## Data Availability

The dataset and code can be accessed through the GitHub repository link: https://github.com/mmc-research-group/tanaka_ml_defect_COMMAT/tree/master.
